# Body size attitudes of 4 year old girls and boys: the role of environmental and individual influences

**DOI:** 10.1186/2050-2974-1-S1-O52

**Published:** 2013-11-14

**Authors:** Emma Spiel, Susan Paxton, Stephanie Damiano, Karen Gregg

**Affiliations:** 1 La Trobe University, Australia

## 

Very little is known about the development of body size attitudes in young children. We examined weight attitudes in 279 4-year-old girls and boys to test the hypothesis that children hold stereotypical beliefs about others based on body size, and that both child and maternal body image variables are related to these attitudes. Children completed an interview in which they were asked to select a figure to represent a child with positive or negative characteristics from an array of figures varying from very thin to large, and also to select figures to represent their own perceived body size, and their ideal body size. Child BMI-for-age z-score, as well as mothers' body dissatisfaction, internalization of the thin ideal and dieting were assessed. Children chose larger figures to represent negative compared to positive characteristics. Maternal body image attitudes were correlated with figure size selection for positive, but not negative characteristics. Preliminary findings show a trend that children's social environments are important in the development of positive body size attitudes.

This abstract was presented in the **Body Image** stream of the 2013 ANZAED Conference.

